# Electron beam fabrication of a microfluidic device for studying submicron-scale bacteria

**DOI:** 10.1186/1477-3155-11-12

**Published:** 2013-04-10

**Authors:** M Charl Moolman, Zhuangxiong Huang, Sriram Tiruvadi Krishnan, Jacob WJ Kerssemakers, Nynke H Dekker

**Affiliations:** 1 Department of Bionanoscience, Kavli Institute of Nanoscience, Faculty of Applied Sciences, Delft University of Technology, Lorentzweg 1, 2628 CJ Delft, The Netherlands

**Keywords:** Microfluidics, Poly(dimethylsiloxane), Electron beam lithography, Micro-nanofabrication, Bacterial growth, *Lactococcus lactis*, *Escherichia coli*

## Abstract

**Background:**

Controlled restriction of cellular movement using microfluidics allows one to study individual cells to gain insight into aspects of their physiology and behaviour. For example, the use of micron-sized growth channels that confine individual *Escherichia coli* has yielded novel insights into cell growth and death. To extend this approach to other species of bacteria, many of whom have dimensions in the sub-micron range, or to a larger range of growth conditions, a readily-fabricated device containing sub-micron features is required.

**Results:**

Here we detail the fabrication of a versatile device with growth channels whose widths range from 0.3 *μ*m to 0.8 *μ*m. The device is fabricated using electron beam lithography, which provides excellent control over the shape and size of different growth channels and facilitates the rapid-prototyping of new designs. Features are successfully transferred first into silicon, and subsequently into the polydimethylsiloxane that forms the basis of the working microfluidic device. We demonstrate that the growth of sub-micron scale bacteria such as *Lactococcus lactis* or *Escherichia coli* cultured in minimal medium can be followed in such a device over several generations.

**Conclusions:**

We have presented a detailed protocol based on electron beam fabrication together with specific dry etching procedures for the fabrication of a microfluidic device suited to study submicron-sized bacteria. We have demonstrated that both Gram-positive and Gram-negative bacteria can be successfully loaded and imaged over a number of generations in this device. Similar devices could potentially be used to study other submicron-sized organisms under conditions in which the height and shape of the growth channels are crucial to the experimental design.

## Background

The use of microfluidics in biological research has gained much popularity in recent years. Subfields that have been impacted by this technology range from tissue engineering [1], cancer stem cell research [2], gene expression of embryonic stem cells [3], protein interactions [4], diagnostic medicine [5] as well as microbial physiology and behaviour [6-8], to name but a few. A specific contribution to the field of microbiology is the ability to observe and manipulate single cells [9]. Individual cells can significantly differ from one another in terms of their biochemistry and genetics [10]. The ability to observe individual cells under controlled conditions provides one with the ability to investigate the individual functioning of cells as well as their mutual behaviour [11-17]. For example, the use of microfluidics has facilitated the study of molecular behaviour inside individual cells, as demonstrated by e.g. Taniguchi *et al.*[18] in their study of protein and mRNA expression at the single-molecule level inside individual living cells. An additional advantage of microfluidics is that it provides one with the ability to observe many more generations than with conventional agarose pads [9].

Recently Wang *et al.*[19] utilized a microfluidic device to quantitatively study steady-state growth and division of individual *Escherichia coli* (*E. coli*) cells at a defined reproductive age grown in Luria-Bertani (LB) medium. Such a device makes it possible to study a large number of cells that inherit the same cell pole over multiple generations. In their design, cells are confined in growth channels oriented perpendicularly to a trench through which growth medium (LB) is flown. The width and height of the channels are similar to the dimensions of *E. coli*, which has a diameter of *ca.* 1 *μ*m and a length of *ca.* 2.5 *μ*m under these conditions [20,21]. Cells are immobilized, in the absence of chemical fixation, at the far end of such a growth channel (*ca.* 25 *μ*m in length). The length of the growth channels is chosen so as to ensure sufficient supply of nutrients to the bacteria by diffusion. Such an immobilization scheme allows one to simultaneously study numerous different cells for extensive periods of time.

A microfluidic device that would allow one to probe smaller microorganisms would greatly enhance the applicability of this approach. Notably, many bacterial species have submicron-scale dimensions for which growth channels would require significantly reduced widths. Examples of such species include e.g. *Mycoplasma* (diameter 0.2−0.4 *μ*m [22]), *Prochlorococcus* (diameter 0.5−0.7 *μ*m [23]), and *Lactococcus lactis* (diameter of *ca.* 0.75−0.95 *μ*m [24]), for which growth channels would require significantly reduced widths. A single device with growth channels of variable widths would furthermore provide maximal flexibility for studying different types of bacteria under a variety of growth conditions. A recent advance along these lines described the fabrication of sub-micron channels in agarose [25]. However, both this approach as well as the device utilized by Wang *et al.* are fabricated using conventional photolithography. While this is a widely available and convenient technique, for the fabrication of devices with smaller dimensions it becomes more cumbersome and alternative approaches such as electron beam lithography (EBL) [26] become more suitable. EBL can readily fabricate smaller features (*ca*. 20 nm in lateral dimensions) compared to conventional photolithography (*ca.* 1 *μ*m) [27], while simultaneously affording greater control of the structure size and shape. An additional advantage of EBL is the reduced time from design to final device, which is convenient in a research environment where it is frequently required to change and improve a device on a relatively short time scale. The structural control and rapid-prototyping needs are thus more easily met by EBL than by conventional photolithography.

**Figure 1:**
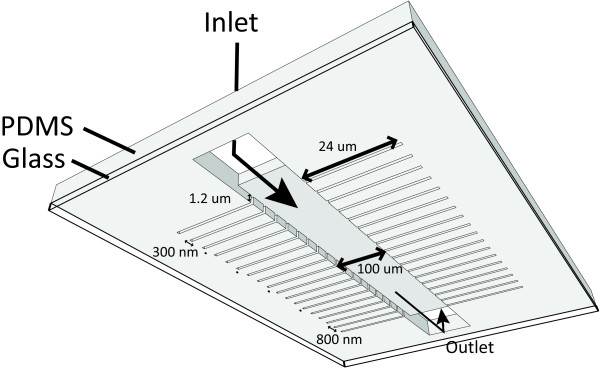
**Schematic overview of the final PDMS device together with bound cover glass (not to scale).** Dimensions are as follows: the main trench has a width of 100 *μ*m and a length of 12.5 mm, growth slits are 24 *μ*m long and 1.2 *μ*m deep, while their widths range from 0.3 *μ*m to 0.8 *μ*m.

Here we present an EBL and poly(dimethylsiloxane) (PDMS) [28] soft-lithography [29] protocol for the fabrication of such a microfluidic device for microbial studies. The device that contains channels of variable widths ranging from 0.3 *μ*m to 0.8 *μ*m (Figure 1), designed to accommodate the typical range of sizes of sub-micron scale bacteria. The channels in the microfluidic device are formed out of PDMS, which has a number of attractive features that makes it an excellent material for fabricating microfluidic devices [27]. For example, sub-micron sized structures down to *ca.* 100 nm are possible in PDMS [30]. We demonstrate the use of the final microfluidic device first by injecting fluorescent dye into the channels and imaging the resulting fluorescence, and then by illustrating how sub-micron sized bacteria such as the Gram-positive *Lactococcus lactis* (*L. lactis*) and the Gram-negative bacterium *E. coli* (grown in minimal conditions) can successfully be loaded into the channels and grown for several generations. To illustrate the power of the device using *E. coli* cells, we follow the division occurrence of the mother cell as function of time. In this experiment eight cell cycles are observed.

## Results and discussion

### Device fabrication

**Figure 2:**
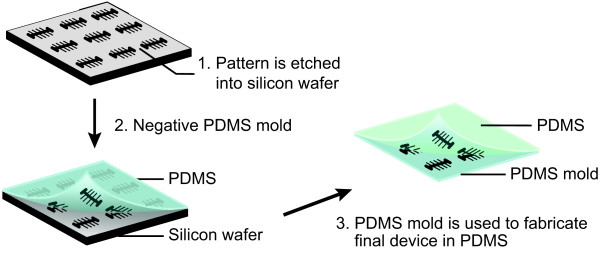
**Schematic overview of the three principal steps. (Step 1)** The patterns are etched into a 4^″^ silicon wafer. **(Step 2)** The silicon wafer is used as a mold to fabricate the negative structures in PDMS. **(Step 3)** The PDMS mold is subsequently used to fabricate the final structures in PDMS. For simplicity we depict only 9 structures (whilst typically a wafer contains 24).

The fabrication of the device contains three principal steps (Figure 2). Firstly, we etch the pattern of the device into a 4^″^ silicon (Si) wafer (Figure 2, Step 1). We accomplish this by employing EBL together with specific dry etching protocols. Secondly, we use PDMS to create a negative mold of the structure that was fabricated in the Si wafer (Figure 2, Step 2). Finally, we make use of this PDMS mold to fabricate the final device in PDMS (Figure 2, Step 3). We note that an alternative approach could involve the fabrication of a negative (as opposed to a positive) Si mold, which would reduce the number of PDMS steps. However, we do not favor such an approach, as it would require one to fabricate the small growth channels *ca.* 25 *μ*m into the Si wafer. To optimize the fabrication yield, we employ a wafer that is much larger than the size of a single device. This allows us fabricate multiple individual devices (in our current protocol 24 in total) in parallel. The only step excepted from the parallel approach is the final bonding step (of glass to PDMS), which is carried out for each device individually. In the following paragraphs we describe the fabrication in Si and PDMS in detail.

**Figure 3:**
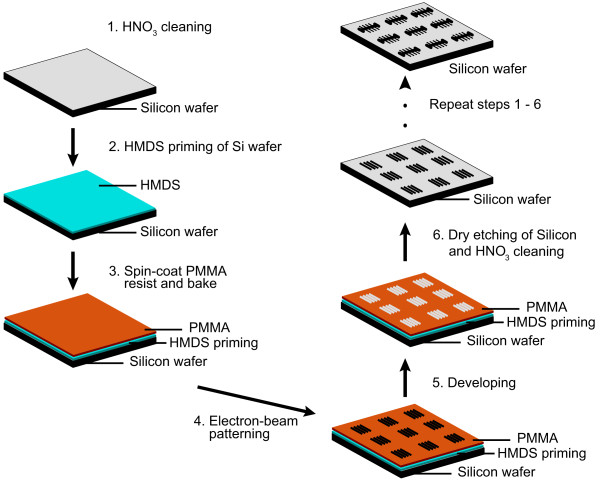
**Schematic of the fabrication of multiple structures in Si with sub-micron size growth channels using EBL and dry etching.** The growth channels are etched first, followed by the main trench. The steps to conduct this are as follows. **(Steps 1–3)** A 4^″^ silicon wafer is cleaned and prepared for electron beam patterning. **(Steps 4–5)** Specific regions of the PMMA is exposed to the electron beam and developed. **(Step 6)** Dry etching of the structures into the wafer is performed. This whole process is then repeated to etch the main trench into the silicon wafer.

The main steps in fabricating the structures in Si are depicted in Figure 3. We fabricate the structures into a 4^″^ diameter Si wafer (University Wafer, USA). We first fabricate the small growth channels, followed by the main trench. In the first step, we ultrasonically clean the wafer in fuming nitric acid (100% HNO_3_) for 15 min (Figure 3, Step 1), rinse in deionized (DI) water, and spin dry. We then subsequently prime the wafer surface for resist adhesion using hexamethyldisilazane (HMDS) by spin-coating at 3000 rpm for 1 min (Figure 3, Step 2). After this, we spin-coat an approximately 2.2 *μ*m thick layer of poly(methyl methacrylate) (PMMA) 950K A11 positive electron-beam resist onto the wafer at 3000 rpm for 1 min and bake it for 2 min at 100°C and for 10 min at 175°C. (Figure 3, Step 3).

The first pattern, i.e. the small growth channels, can now be written into the wafer (Figure 3, Step 4). We make use of a Leica EBPG 5000+ (acceleration voltage 100 kV, aperture 400 *μ*m) to write the pattern on the wafer. Here we use a spot size of *ca.* 25 nm and a current of *ca.* 46 nA. We choose the beam step size (BSS) to be 20 nm and the dose 1400 *μ*C/cm^2^.

Following electron beam exposure, we develop the exposed PMMA (Figure 3, Step 5) by using methyl isobutyl ketone (MIBK) and isopropyl alcohol (IPA). We place the wafer in a beaker containing a 3:1 ratio of IPA and MIBK for 60 s. Directly afterwards, we place the wafer in a beaker containing IPA only for 30 s, and subsequently spin it dry. We then clean the wafer by exposing the wafer to an O_2_ plasma in a microwave plasma system (Tepla 100) with the power set to 100 W and the pressure maintained at approximately 0.15 mbar.

Following PMMA development, we perform the dry etching of the growth channels (Figure 3, Step 6). This is achieved by using an inductive coupled plasma (ICP) reactive-ion etcher (RIE) (Adixen AMS 100 I-speeder) with a mixture of 15 sccm sulfur hexafluoride(SF_6_), 20 sccm octafluorocyclobutane(C_4_F_8_), 10 sccm methane(CH_4_) that is diluted in 100 sccm helium (He). We set the ICP power to 2000 W, and the capacitive coupled plasma (CCP) power (biased power) to 250 W. We maintain the sample holder at 0°C during the entire process. The wall of the main chamber is maintained at 200°C to inhibit polymer deposition there. We fix the sample holder height at 200 mm and maintain a low pressure of *ca.* 1 Pa. We perform this etching process for 3 min. At an etching rate of *ca.* 390 nm/min, this results in approximately 1.2 *μ*m deep growth channels.

**Figure 4:**
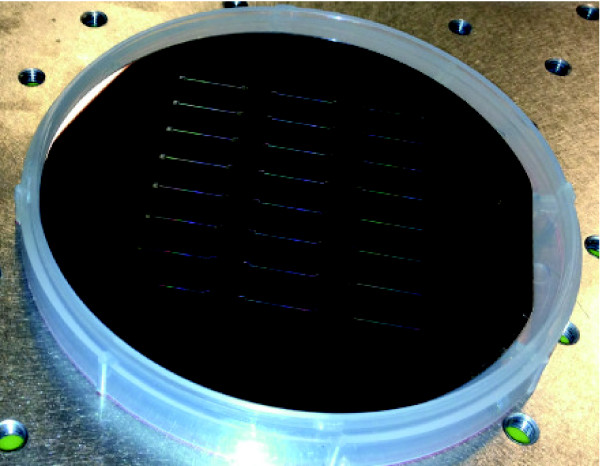
**Image of the final Si wafer following the fabrication.** One can clearly observe the 24 structures etched into the Si wafer. Here the main trench as well as inlet outlet ports are clearly visible. The side growth channels are too narrow to be visualized in this manner.

Next we fabricate the main trench together with the inlet and outlet using an identical procedure save for two aspects. Firstly we perform the patterning with a larger spot size, *ca.* 113 nm, at a current of *ca.* 193 nA and an increased BSS of 100 nm. Secondly, we make use of the dry etching process known as Bosch deep reactive ion etching (DRIE) [31]. This type of etching is different than the previous method in the way that the etching process consists of repeating etching (SF_6_) and passivation cycles (C_4_F_8_). The passivation step ensures that the sidewalls of the structure being etched are protected during the etching process (see references [31,32] for a more thorough description). We maintain the sample holder at 10°C during the entire etching process. We keep the pressure at approximately 0.04 mbar. We perform the etching step with 200 sccm SF_6_ for 7 s with the ICP power 2000 W and the CCP power off. We execute the passivation step with 80 sccm C_4_F_8_ for 2 s with the ICP power 2000 W and the CCP power set in chopped low frequency (LF) bias mode: 80 W, ON 10 ms, OFF 90 ms. We repeat this etching cycle for 5 min. The etching rate for Si is approximately 5 *μ*m/min, which for these settings results in the *ca.* 25 *μ*m deep trench. After this, we again clean the wafer in 100% HNO_3_ for 15 min, spin it dry and rinse with DI water. A sample wafer following all fabrication steps is depicted in Figure 4.

**Figure 5:**
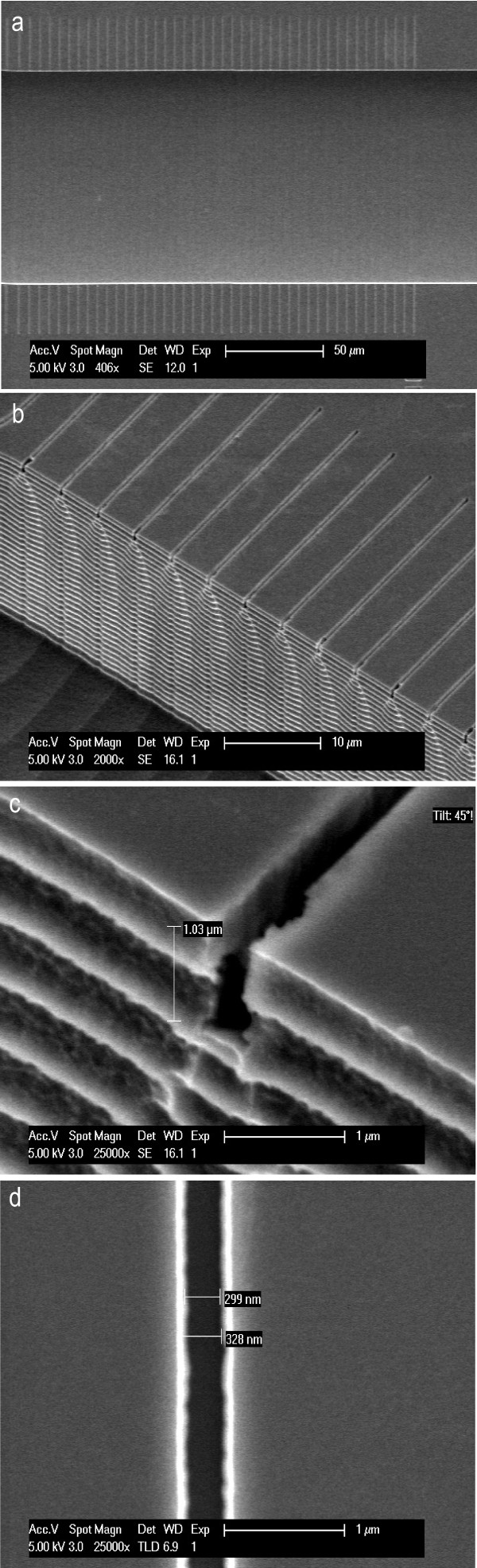
**SEM images of fabricated sub-micron channels in silicon. (a)** Top overview of a part of the main trench and the small growth channels. For illustration purposes we only show the small channels, but obtained similar results for the bigger growth channels. Here one clearly observes the control of the etching process as shown by the sharp boundaries of the structures. **(b)** Side view of a part of the main trench and the small growth channels. It is evident from this image that the etching resulted in the appropriate structures. (**c**,**d**) Zooming in on one of the small channels to illustrate its dimensions. The scalloping effect seen in (b) and **(c)** has to do with the repeating passivation and etching cycles of the Bosch process.

We validate the fabrication of the structure in Si by SEM using a FEI/Philips XL30S/FEG. Four different SEM images are shown in Figure 5. A top and side view of a portion of the main trench together with the smallest growth channels (i.e. 0.3 *μ*m width) are shown in (Figure 5a,b). As can be seen from the images, the etching process is successful and we maintain good control of the structures. The scalloping effect that is visible at the side of the trench is a result of the Bosch DRIE etching process. Zooming in on one of the small channels, we observe that the height and depth correspond to the expected values (Figure 5c,d). This implies that the PMMA provides sufficient protection during the etching process. We note that shallower channels could be fabricated simply by reducing the etching time.

The final step we perform before the wafer can be used as a mold is a silanization step. This is necessary to reduce the adhesion between PDMS and Si in the curing step and is achieved as follows. We expose the wafer to an O_2_ plasma for 10 s. We then immediately place the wafer in a desiccator together with 15 *μ*L of silanizing agent (tridecafluoro-1,1,2,2-tetrahydrooctyl trichlorosilane) (TFOCS) [33]. We place the desiccator under a vacuum, which results in evaporation of the silanizing agent and formation of a monolayer on the surface of the Si wafer. This layer renders the Si wafer extremely hydrophobic, preventing the PDMS from adhering to it. After 2 hours under vacuum, the Si wafer is ready to be used as a mold.

**Figure 6:**
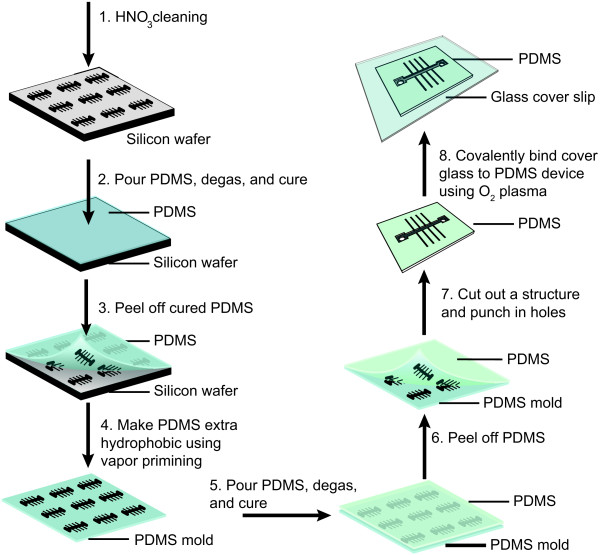
**Schematic of the fabrication of multiple structures in PDMS with sub-micron sized growth channels. (Step 1)** The wafer is cleaned with HNO_3_. **(Step 2–3)** A 1:5 ratio PDMS is poured on the wafer, cured, and carefully peeled off. **(Step 4)** A silanization step is performed on the resulting PDMS mold. **(Step 5)** A 1:10 ratio PDMS is poured on the PDMS mold and cured. **(Steps 6)** The two PDMS layers are carefully separated from one other. **(Step 7)** A single structure is cut out, and an inlet and outlet port are punched. **(Step 8)** PDMS and a clean cover glass are bound together using O_2_ plasma.

To fabricate the final PDMS microfluidic device, we first utilize the Si wafer as a mold to fabricate a PDMS negative of the structures. We then subsequently use the PDMS negative mold to fabricate the final structures in PDMS. A total of eight principal steps yield the final microfluidic device in PDMS (Figure 6).

In order to fabricate the structures in PDMS we perform the following steps. If the Si wafer is stored for longer than 24 h, we first ultrasonically clean it in 100% HNO_3_ for 15 min, rinse with DI water and spin it dry (Figure 6, Step 1). Secondly, we prepare PDMS (Mavom Chemical Solutions DC Sylgard 184 elastomer kit) by mixing an elastomer base and curing agent in a ratio of 1:5 to obtain a relatively stiff mold. Afterwards we mix the PDMS thoroughly, we pour it over the clean Si wafer and degas it in a desiccator (Figure 6, Step 2). We subsequently bake the PDMS and Si wafer for 2 h at 85°C, and afterwards leave it to cool down for *ca.* 30 min. In the final step, we carefully peel off the PDMS from the Si wafer (Figure 6, Step 3).

**Figure 7:**
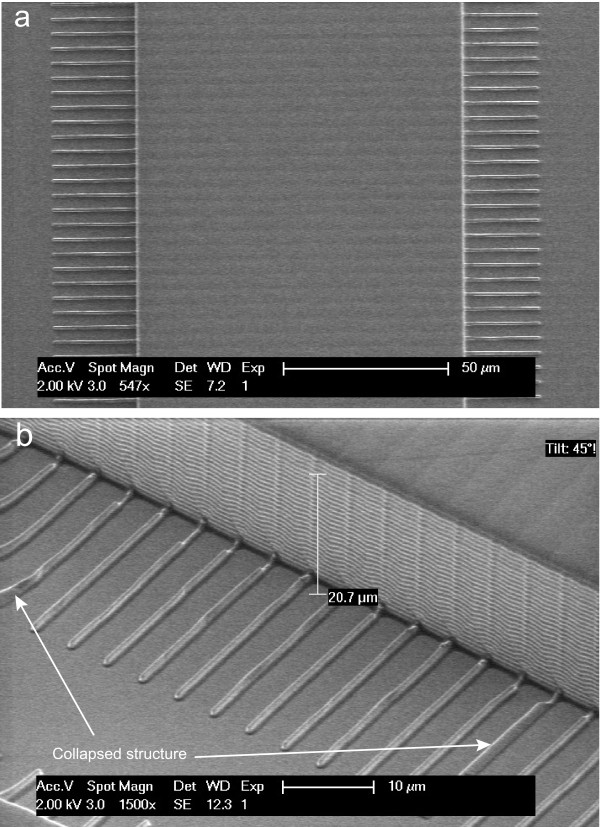
**SEM image of PDMS mold with a thin layer of gold on its surface. (a)** A top view of the biggest channels. **(b)** A side view of the smallest channels. In both images it is clear that the structures are successfully replicated from silicon into PDMS.

To verify the successful replica of the structure onto PDMS, we investigate the structure with a SEM. Before we perform the SEM imaging, we first coat the PDMS with a thin layer of gold (Au) to avoid charging during the imaging process. SEM images are made using a FEI/Philips XL30S/FEG (Figure 7). One can observe from the top view of the largest growth channels (Figure 7a) and a side view of the smallest channels (Figure 7b), that the structures are successfully created in PDMS. A small subset of the growth channels suffers from collapse (Figure 7b, white arrows), likely the result of the relatively high aspect ratio of the channels (0.3 *μ*m : 1.2 *μ*m) in combination with the relative softness of PDMS. As mentioned above, we can reduce the depth of the growth channels by etching for a shorter time, which could potentially increase the yield. A different approach to circumvent channel collapse would be to utilize a composite two-layer process consisting of a thick “typical” PDMS layer and a thin *h*-PDMS (“hard” PDMS) layer [34]. This approach has been shown to increase the yield when fabricating sub-100 nm size structures using soft lithography. In our current protocol, however, there are enough upright channels that can serve as a mold for the next step, so we proceed without further alterations. We perform a further silanization process (Figure 6, Step 4), to reduce the adhesion between the two PDMS layers after the curing process.

Finally we fabricate the positive structures in PDMS. We mix PDMS in a 1:10 ratio, degas, and pour it onto the previously cured PDMS mold (Figure 6, Step 5). We then again degas and allow to cure for 2 h at 85°C. Once the curing is complete, we leave the PDMS to cool down for at least 30 min. Subsequently we carefully separate the two PDMS layers from each other (Figure 6, Step 6). At this point the PDMS mold can be stored for later use.

The cured PDMS layer contains 24 positive structures, each of which can be used in an experiment. To study an organism under the microscope utilizing the device, the PDMS device should have an inlet and outlet port for media exchange, as well as a cover glass that seals the device. To fabricate the inlet and outlet ports we first carefully cut out a single PDMS device and punch holes at the two sides of the main trench using a 0.75 mm Harris Uni-Core puncher (Figure 6, Step 7). To bind the cover glass to the PDMS, we simultaneously expose the clean cover glass (ultra-sonicated in acetone and IPA) and the PDMS devices to an O_2_ plasma using a microwave plasma system (Plasma-Preen I, Plasmatic Systems Inc.) (Figure 6, Step 8). We then bring the two exposed surfaces into contact and press slightly. It is believed that when the surfaces are exposed to plasma, silanol groups (−OH) are developed, which form covalent siloxane bonds (Si −*O*−Si) when the two surfaces are brought into contact [35,36]. We then bake the PDMS and attached cover slips for *ca.* 30 min at 85°C, after which they are ready to be used in an experiment. In the following section we demonstrate the utilization of this device in two types of experiments.

### Utilizing the PDMS device

**Figure 8:**
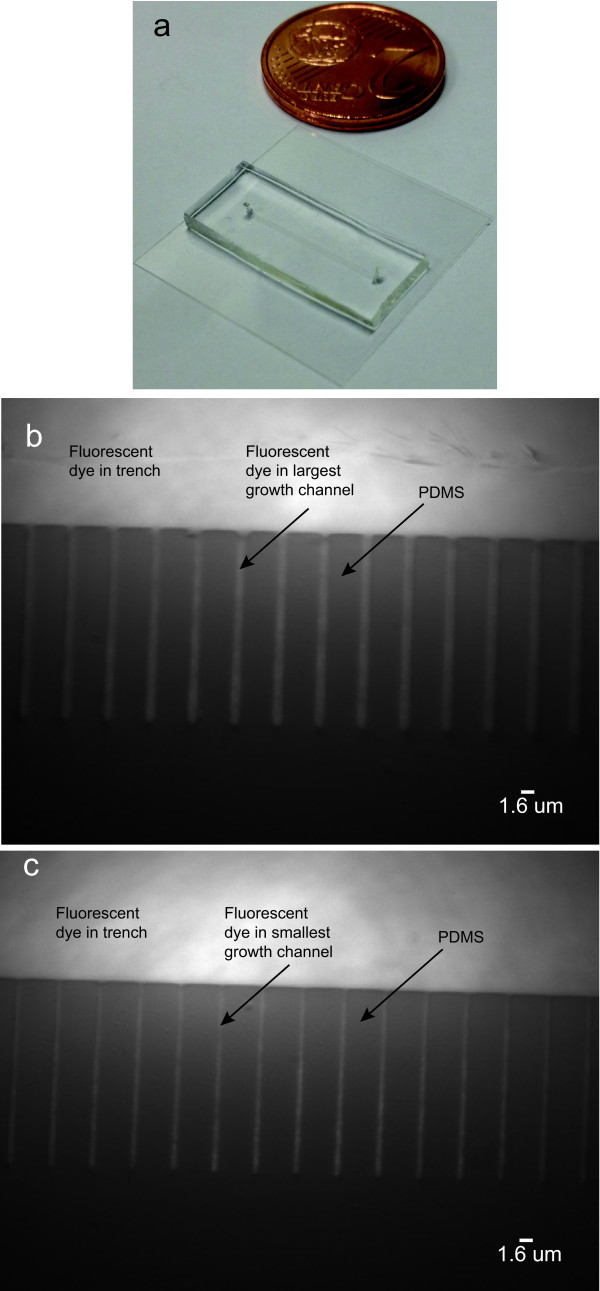
**An example of the final PDMS device as well as images of a fluorescent dye inside the device. (a)** The actual final PDMS device as is used in experiments. **(b)** Presence of fluorescent dye in largest channel, nominally 0.8 *μ*m, **(c)** Presence of fluorescent dye in smallest channel, nominally 0.3 *μ*m. One observes that the dye is able to enter the growth channels. The inhomogeneity of the light intensity observed is due to the Gaussian profile of the laser beam. We performed a control experiment (data not shown here) by measuring the fluorescence signal in the absence of the fluorescent dye. During these conditions no significant fluorescence signal was detected in the trench or growth channels. This observation supports our conclusion that the signal detected as shown in the above images are due to the fluorescent dye in the trench and growth channels.

We can now prepare our finished microfluidic device (Figure 8a) for studies of bacterial growth. Firstly we verify that the growth channels can indeed be wetted. We accomplish this by injecting a fluorescent dye into the device, and imaging the resulting fluorescence using a fluorescence microscope. In a distinct device, we demonstrate that bacteria can be loaded into the growth channels. We perform two types of experiments, one in which we inject the Gram-positive bacterium, *L. lactis*, into the device, and in the other experiment where we inject the Gram-negative bacterium, *E. coli*. In both the cases, the cells fit well into the respectively sized channels (*ca.* 0.8 *μ*m) and also subsequently grow and divide.

We illustrate the functionality of the microfluidic devices by injecting a fluorescent liquid (Invitrogen Alexa Fluor 514 Goat Anti-Rabbit IgG 2 mg /mL) into the growth channels. First, we attached tubing to the inlet and outlet of the device. We inject phosphate buffered saline (PBS) (Sigma, 0.01 M PBS - NaCl 0.138 M, KCl 0.0027 M, pH 7.4), into the device. After this, we simultaneously autoclave (120°C for 15 min) the device and tubing. This is done both to ensure sterile conditions when working with micro-organisms and to remove any air bubbles present inside the device. After the autoclaving process is complete, we flush through 50 *μ*L bovine serum albumin (BSA) (10 mg/mL New England Biolabs) through the device and allow it to incubate for at least 15 min. This surface passivation step is done to reduce unwanted sticking of the specimen being studied to the glass and PDMS surfaces. After this incubation period we injected the dye (diluted 1:50 in PBS) into the device and image on a fluorescence microscope.

We successfully wet the growth channels as shown in Figure 8b,c. For illustration purposes we show only the largest growth channels, *ca.* 0.8 *μ*m (Figure 8b) and smallest ones *ca.* 0.3 *μ*m (Figure 8c). One can clearly observe that dye was successfully injected into both types of channels and can readily visualize their differences in size.

Next we demonstrate that sub-micron size bacteria can successfully be observed in the microfluidic device. For this purpose we use both *L. lactis* and *E. coli*. *L. lactis* has a diameter of *ca.* 0.8 *μ*m [24], as do *E. coli* when cultured in a minimal medium [20]. It is thus possible to immobilize both these species of bacteria in the largest growth channel of this type of microfluidic device.

**Figure 9:**
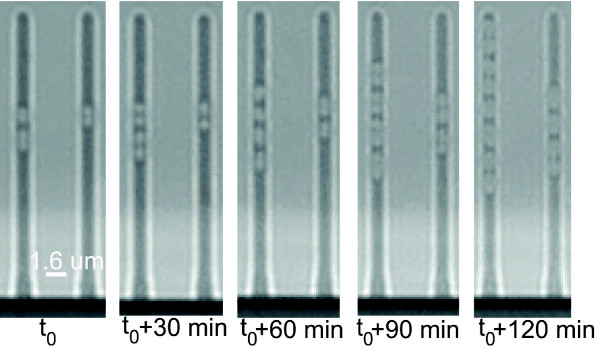
**Time points (every 30 min starting at *t***_**0**_**) of a 2 h time series measurement of *L. Lactis *in the microfluidic device.** Measurements are performed at room temperature (*ca.* 23°C). One can clearly observe growth and division during these two hours. Looking at the first slit, for example one observes the initial two cells becoming 8 cells in 2 h.

We verify the successful growth of *L.lactis* in the device as follows. We inject *L. lactis* into the device and load them into the growth channels by means of centrifugation (Figure 9). The centrifugation step itself increases the loading efficiency of bacteria into the growth channels by a factor of three. The cells depicted are in the largest size growth channels (*ca.* 0.8 *μ*m), as can be expected given the nominal size of *L. lactis*. During the course of the experiment the cells remain essentially immobilized. Bright field microscopy is used to image the cells. Here we show example time points of approximately 30 min intervals as to demonstrate the growth of *L. lactis* over time. One can clearly observe cell growth in the sample channels shown. As is evident from these time points, the cells grew for approximately two generations during the 2 h measurement duration. The doubling time of these bacteria is estimated from this measurement to be *ca.* 60 min under these conditions.

**Figure 10:**
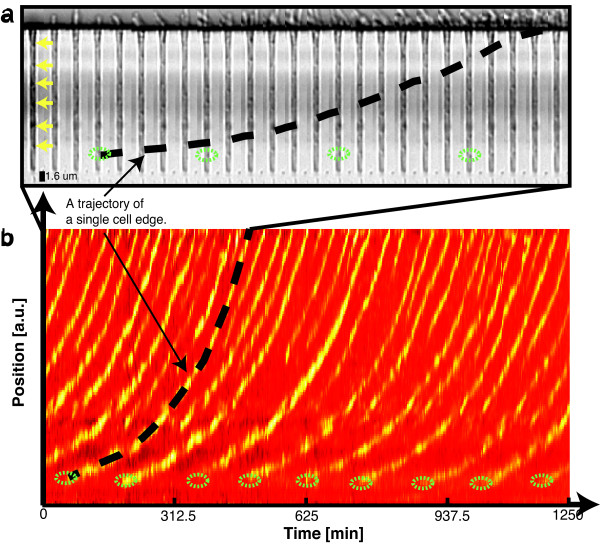
**A montage (a) and kymograph (b) of *E. coli *growing in the microfluidic device. (a)** A montage (interval of 25 minutes) made of a single growth channel using the *ImageJ* software package. The yellow arrows in the montage indicate the edges of the different bacterial cells which corresponds to the yellow traces seen at the beginning of the kymograph. **(b)** A kymograph (with a duration of *ca.* 21 hrs) of the corresponding growth channel is depicted here. The yellow traces indicate the position of cell edges over time. Custom *Matlab* software was written and used to construct the kymograph from the bright-field images. The dotted black line in both **(a)** and **(b)** is drawn by hand and illustrates our ability to follow the spatial movement of a specific bacterium edge as function of time. The division occurrence of the mother cell at the bottom of the growth channel is highlighted by a green ellipse in both the montage as well as the kymograph. From the kymograph it is clear that the growth and division of a single mother cell at the far end of the growth channel can be quantified over multiple cell cycles. In this experiment we observe eight mother cell divisions.

As with the *L. lactis* cells, we successfully observe *E. coli* growth over multiple generations (Figure 10) in the growth channels of the micro-fluidic device. Figure 10a depicts a montage (25 min intervals) of a single growth channel, made using the *Make Montage* function in the *ImageJ* software package. Cell growth is clearly visible in the 625 min fragment, as well as the division of the mother cell (namely four divisions) at the far end of the growth channel. Despite the relatively low contrast of bright field images, we are able to accurately follow the edges of bacteria, and thus track the division process of single cells over time. This is graphically illustrated by means of the dashed black line in the montage. Figure 10b is a kymograph of the same growth channel. The yellow traces in the kymograph indicate the position of cell edges over time. The yellow arrows in the montage highlight the edges of the bacteria which corresponds to the yellow traces seen at the beginning of the kymograph. We graphically emphasize the edge detection and tracking of a single cell pole by means of the dashed black line that corresponds to the dashed line in the montage. It is thus possible to track a cell edge as function of time until it exits the channel. We can follow, highlighted here by means of green ellipses, the division of the mother cell for numerous cell cycles, which is in essence the advantage of using this type of device over conventional agarose pads. In this experiment eight cell cycles of the mother cell are observed. The average doubling times that we observe are found to be independent of a cell’s position within the growth channel.

## Conclusions

We have presented a detailed protocol based on EBL together with specific dry etching procedures for the fabrication of a microfluidic device suited to study submicron-sized bacteria. In comparison to approaches based on conventional optical lithography, our method provides enhanced versatility and control of the dimensions of the growth channels while satisfying the rapid-prototyping needs in a research environment. The widths of the submicron growth channels allow for the potential immobilization and study of different size bacteria with widths ranging from 0.3 *μ*m to 0.8 *μ*m. We verified by means of SEM that these structures are successfully transferred from Si into PDMS as well as from PDMS into PDMS. As a proof-of-principle, we demonstrated that both Gram-positive and Gram-negative bacteria can successfully be loaded and imaged over a number of generations in this device. Similar microfluidic devices could potentially be used to study other submicron-sized organisms under conditions in which the height and shape of the growth channels are crucial to the experimental design.

## Methods

### Microscopy

The microscope setup used during the experiments consists of a commercial Nikon Ti, a customized laser illumination path, and a personal computer (PC) running Nikon NIS elements. Different illumination schemes were used for the different measurements. A Cobolt Fandango 515 nm continuous wave (CW) diode-pumped solid-state (DPSS) laser is used to excite the fluorescent dye to verify device wettability. The experiments with *L.lactis* and *E. coli* are performed using standard brightfield illumination. In all the experiments a Nikon CFI Apo TIRF 100x oil (NA 1.49) objective is used for imaging. Bright field images are acquired every 5 minutes.

### Cell culture preparation for microscopy

The *L.lactis* cultures are grown directly from plate in Luria-Bertani medium (LB) at 30°C until an OD _600_≈0.2 is reached. The cell culture is then concentrated by centrifugation and injected into the PDMS device. A syringe pump is used to inject fresh LB medium.

*E. coli* are grown in M9 medium supplemented with 0.3*%* glycerol at 37°C overnight with shaking, and sub-cultured in the morning until an OD _600_≈0.2 is reached. The cell culture is then concentrated by centrifugation and injected into the PDMS device. A syringe pump is used to inject fresh M9-glycerol medium.

## Competing interests

The authors declare that they have no competing interests.

## Authors’ contributions

MCM, ZH and NHD designed the research. ZH and MCM developed and performed the fabrication procedures. MCM and STK performed the microscopy experiments. JWJ and MCM wrote the software and analyzed the data. MCM, ZH and NHD wrote the manuscript. All authors have read and approved the final manuscript.
